# Unraveling the intestinal epithelial barrier in cyanotoxin microcystin-treated Caco-2 cell monolayers

**DOI:** 10.1111/nyas.14870

**Published:** 2022-07-26

**Authors:** Jan-Leo Kaak, Fábia D. Lobo de Sá, Jerrold R. Turner, Jörg-Dieter Schulzke, Roland Bücker

**Affiliations:** 1 Department of Gastroenterology, Infectious Diseases and Rheumatology, Clinical Physiology/Nutritional Medicine, Charité – Universitätsmedizin Berlin, Berlin, Germany; 2 Laboratory of Mucosal Barrier Pathobiology, Department of Pathology, Brigham and Women’s Hospital and Harvard Medical School, Boston, Massachusetts, USA

**Keywords:** actin, apoptosis, claudin, MLCK, PIK, tight junction

## Abstract

Microcystin is a widespread cyanobacterial toxin that affects the intestine to produce diarrheal symptoms after ingestion of freshwater blue-green algae. Our study aimed to characterize the mechanism by which the toxin leads to diarrhea via epithelial barrier dysfunction in a small intestine Caco-2 cell model. Microcystin-treated human Caco-2 epithelial monolayers were functionally and molecularly analyzed for barrier dysfunction. Tight junctions (TJs) and cell damage were analyzed in relation to transepithelial electrical resistance (TER) changes. TER of microcystin-treated Caco-2 cells was reduced by 65% of the initial value after 24 h; concomitantly, permeability for fluorescein increased 2.6-fold. Western blot analysis showed reduced claudin-1 expression, while expression of claudin-3 and −4 remained unchanged. Super-resolution stimulated emission depletion microscopy revealed that TJ integrity was compromised by fraying and splitting of the TJ domain of the epithelial cells. Epithelial apoptosis did not significantly contribute to epithelial barrier dysfunction, while cytoskeletal actomyosin constriction was associated with TJ disintegration and the barrier defect. Our results indicate that microcystin causes intestinal barrier leakiness, which helps to explain the leak flux type of diarrhea as the main pathomechanism after ingestion of cyanobacterial toxin.

## INTRODUCTION

Hazardous algal blooms formed by cyanobacteria pose an emerging threat to the health of humans and animals, such as birds, mammals, and the aquatic life.^1^ For humans, poisoning frequently causes gastrointestinal symptoms. This occurs after ingestion of surface freshwater from lakes contaminated with cyanobacterial toxins during blooms of blue-green algae. When contaminated water is swallowed, short-term illness is associated with symptoms, such as stomach pain, nausea, vomiting, and diarrhea. In rare cases, it can lead to fatal liver failure after large amounts are ingested. Long-term exposure to cyanobacterial toxins may lead to the development of liver cancer.^2^ Cyanotoxins, in particular microcystins, are mainly isolated from *Microcystis aeruginosa*, but also other *Microcystis* spp. and additional cyanobacterial genera. These toxins have been shown to act as hepatotoxins and have been proposed to be inducers of gastrointestinal disorders.^3,4^ However, the diarrheal mechanism of the common cyanotoxin microcystin, and its action on the epithelial transport and/or barrier function in the small intestine, remain unclear.

The aim of our study here was to evaluate the involvement of possible barrier dysfunction as the diarrheal mechanism from microcystin poisoning. As a main structure for epithelial barrier function, we examined the important barrier-forming claudins of the tight junction (TJ). TJ integrity changes and epithelial cell death events have been discussed to be responsible for the diarrheal outcome after microcystin ingestion.

One of the affected target organs of the toxins is the upper gastrointestinal tract, where uptake of microcystin in the small intestine occurs through the apical cell membranes both receptor-independently and via organic anion transporting polypeptide receptor–dependent uptake mechanisms in liver cells.^5^ Microcystins are cyclic heptapeptides that exhibit toxic effects based on the inhibition of protein phosphatases PP1 and PP2A.^6^ Surprisingly, other toxins from marine dinoflagellates, such as okadaic acid, have different structures yet also affect PP1 and PP2A.^7^ After uptake of these toxins, target cells experience overphosphorylation that causes cytopathic effects via cytoskeleton alterations and, ultimately, cytotoxic outcome.^8^ It is conceivable that direct phosphorylation of TJ proteins leads to increased stability of TJ proteins in the membrane and enhances barrier function; alternatively, phosphatase inhibition may potentiate signaling by kinases, for example, myosin light-chain kinase (MLCK), that triggers cytoskeletal actomyosin constriction and negatively affects TJ integrity and the epithelial barrier.

In the present study, we characterized the possible mechanisms, such as leak flux induction by disruption of the intestinal epithelial barrier with respect to the modulation of barrier-maintaining claudins in a small intestinal Caco-2 cell model.

## MATERIALS AND METHODS

### Cell culture

The human colon carcinoma cell line Caco-2 was cultured in minimum essential media – with Glutamax-1 supplementation with 15% fetal calf serum and 1% penicillin-streptomycin (all: Gibco, Life Technologies, Carlsbad, CA, USA) in 25 cm^2^ cell culture flasks (Corning, Corning, NY, USA) at 37°C and 5% CO_2_. Cells were fed three times per week for 14–21 days and were then seeded on Millicell PCF filters (0.4 μm pore size; Merck Millipore, Darmstadt, Germany). Experiments started 21 days after seeding when the cells developed small intestine-like properties and reached confluency.

### Toxin treatment

Microcystin-LR (MC-LR, with amino acids leucine [L] and arginine [R] at positions 2 and 4 [Enzo Life Sciences, Farmingdale, NY, USA]) treatment was performed by medium change. MC-LR stock was dissolved in 100% methanol. Stock solution was added to the medium resulting in a final concentration of 50 μM of MC-LR and 0.5% methanol.

### Epithelial barrier function

The measurement of transepithelial electrical resistance (TER) of the cell monolayer was performed with chopstick electrodes and a Volt–Ohm meter (EVOM3; World Precision Instruments, Sarasota, FL, USA) under sterile conditions at 37°C. Measured TER values were corrected for the resistance of the empty filter and the growth area of 0.6 cm^2^. Measurements of macromolecule fluxes of fluorescein (9 Å; 332 Da) (Sigma Aldrich, St. Louis, MO, USA) from apical to basolateral compartment were performed in 12-well plates. Samples were taken every 15 min from the basal side. Fluorescence was measured in a spectrophotometer (Tecan GmbH, Maennendorf, Switzerland) and permeability of fluorescein was calculated by flux over concentration differences.

### TJ protein expression by Western blot

Changes in TJ protein expression were quantified by Western blot analysis. Cells were washed twice with ice-cold phosphate-buffered saline (Gibco, Life Technologies) and lysed with whole cell lysis buffer containing 150 mM NaCl, 10 mM Tris buffer (pH 7.5), 0.5% Triton X-100, 1% SDS, and Complete Protease Inhibitor (Roche AG, Manheim, Germany). Lysed cells were scraped from the filters, transferred to reaction tubes, and incubated for 30 min on ice with vortexing in between and centrifuged afterward for 30 min at 15,000 × *g* at 4°C. Proteins were loaded on 12.5% polyacrylamide gels. Primary antibodies claudin-1 (1:1000; Invitrogen, Carlsbad CA, USA), claudin-3 (1:1000; Invitrogen), claudin-4 (1:1000; Invitrogen), and, as loading control, *β*-actin (1:10,000; Sigma Aldrich) were incubated over night at 4°C. The following day, peroxidase-conjugated secondary antibody, either goat anti-rabbit IgG or goat anti-mouse IgG (Jackson ImmunoResearch, Ely, UK), was incubated for 2 h at room temperature. Detection of proteins was performed by membrane incubation with SuperSignal West Pico PLUS Stable Peroxide Solution (Thermo Scientific, Waltham, MA, USA). Visualization of proteins was by the Fusion FX imaging system and Fusion FX6 Edge software (Vilber Lourmat Deutschland GmbH, Eberhardzell, Germany), and densitometry was performed using ImageJ software.

### Immunostaining and stimulated emission depletion microscopy of TJ proteins

Epithelial cells grown on filters were washed with PBS and fixed with 2% paraformaldehyde (PFA; Electron Microscopy Science, Hatfield, PA, USA). Primary antibodies were claudin-1 (1:50; Invitrogen), claudin-3 (1:100; Invitrogen), claudin-4 (1:100; Invitrogen), and ZO-1 (1:100; BD Biosciences, Franklin Lakes, NJ, USA) and secondary antibodies were conjugated to Aberrior STAR RED and Aberrior STAR ORANGE (1:200; Abberior GmbH, Göttingen, Germany), F-actin was stained using Phalloidin Abberior STAR RED (1:100; Abberior GmbH) and incubated 105 min at 37°C. After incubation of the cell monolayers, filters were washed (3x PBS, 1× destilled water) and placed on slides. Mounting medium (Abberior GmbH) was heated to 65°C. Mounting medium (25 μl) was placed on each filter and then, a stimulated emission depletion (STED)–compatible cover slide (Carl Zeiss, Jena, Germany) was placed on each filter; two filters were mounted per slide. TJ protein localization was visualized using a STED microscope (STED Abberior Facility Line, Abberior GmbH).

### Epithelial cell death

Apoptotic events were detected by terminal deoxynucleotidyl transferase dUTP nick-end labeling (TUNEL) assay (In situ Cell Death Detection Kit, Roche, Mannheim, Germany), performed according to the manufacturer’s instructions. Nuclei were stained with 4′−6-diamidino-2-phenylindole (DAPI; 1:1000; Roche, Basel, Switzerland). Apoptotic events were visualized using confocal laser-scanning microscopy (CLSM; Zeiss LSM510). Apoptotic TUNEL-positive cells and all nuclei in a low-power field were counted to calculate the ratio of apoptosis. The number of apoptotic events was estimated in five low-power fields per sample.

### Inhibitor studies

Three-week-old Caco-2 cells grown on filter supports were placed in a 24-well plate. Plain medium was used as control. MLCK inhibition groups received medium containing the specific MLCK pseudosubstrate inhibitor D-reverse PIK (DrPIK, 200 μM).^9^ Medium was added 30 min prior to incubation with 50 μM of MC-LR. In further experimental series, the pan-caspase inhibitor Q-VD-OPh hydrate (10 μM; Calbiochem, San Diego, CA, USA) dissolved in DMSO was applied. Solute controls were performed with methanol or DMSO.

### Statistical analysis

All data are presented as mean values ± standard error of the mean, and statistical analysis was performed using GraphPad Prism software using Student’s *t*-test with Welch’s correction for comparison of two groups. *p* < 0.05 was considered to be statistically significant.

## RESULTS

### Epithelial integrity is impaired by microcystin

**FIGURE 1:**
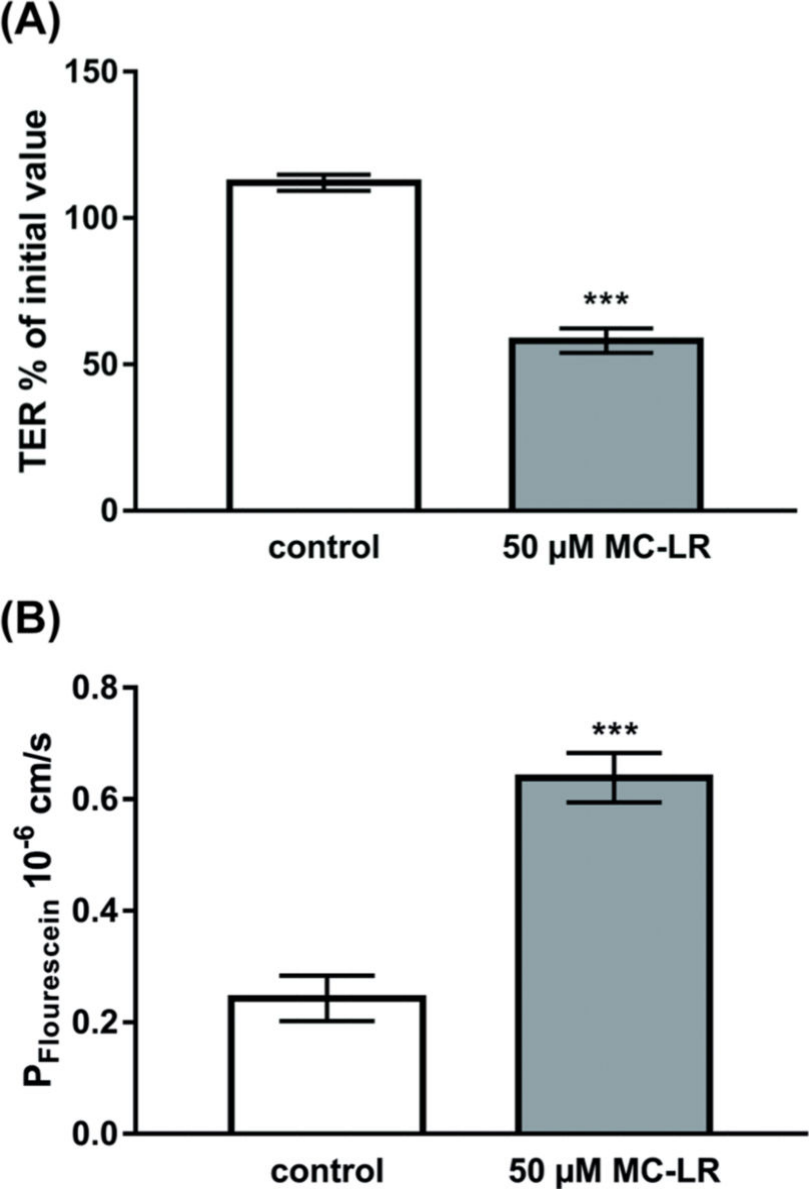
Epithelial barrier function in Caco-2 monolayers 24 h after microcystin-LR treatment. (A) Small intestine-like Caco-2 cell monolayers were measured for TER changes after treatment with microcystin-LR (MC-LR), *n* = 19–24, and (B) the permeability for fluorescein was assessed 24 h after treatment, *n* = 8, ***p < 0.001, *t*-test with Welch’s correction.

The pathogenic impact of MC-LR with respect to barrier breaking of intestinal epithelia was analyzed by treatment of Caco-2 cell monolayers and TER measurements over 24 h; at 4 h after treatment, TER values range from 94% to 85% or 95% to 76% of the initial values with 50 or 100 μM MC-LR, respectively. Thereafter, TER further declined to a pronounced TER drop at 24 h. Fifty micromoles MC-LR reduced TER to half of the initial value after 24 h (Figure 1A). Concomitantly, epithelial permeability for the 9 Å paracellular flux marker molecule fluorescein more than doubled (Figure 1B).

### Barrier-forming claudin-1 is downregulated, and TJs are disrupted

The barrier effect of MC-LR may be due to epithelial leaks resulting from cytotoxic effects on epithelial cells, but it can also be based on disseminated TJ changes. Altered expression of barrier-forming claudins could be a potential target of MC-LR.

**FIGURE 2:**
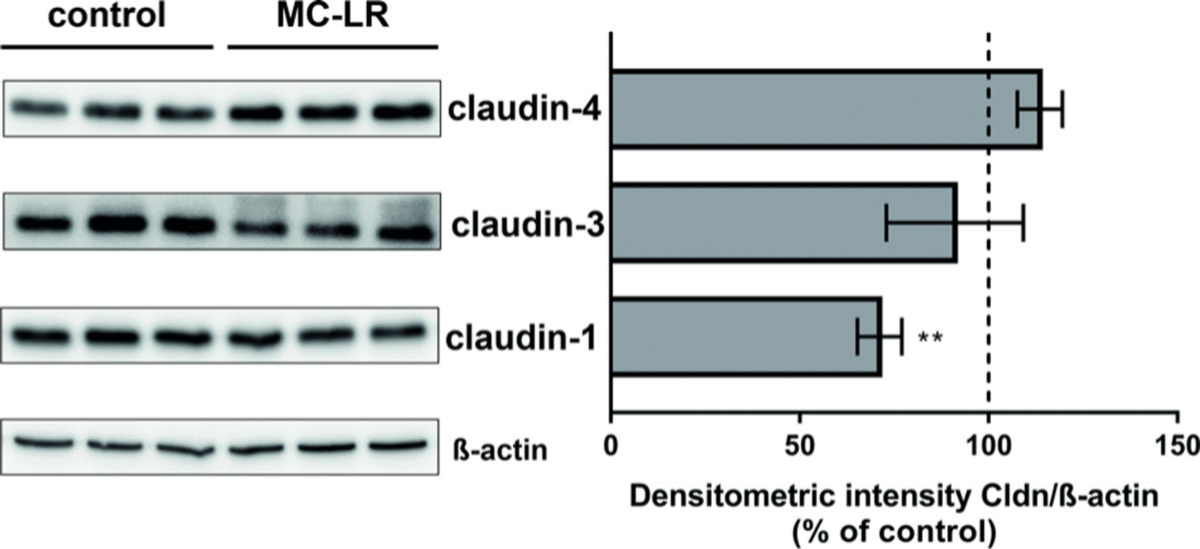
Claudin-1, −3, and −4 protein expression levels in Caco-2 monolayers 24 h after microcystin-LR treatment. Changes in protein expression were assessed by densitometry in Western blots from small intestine–like Caco-2 cell monolayers after 24 h of treatment with MC-LR; *n* = 4, **p < 0.01, *t*-test with Welch’s correction.

In densitometric analysis of Western blots from MC-LR-treated Caco-2 cells, protein expression of claudin-1 was reduced by 29% (Figure 2), while the expression of claudin-3 and −4 remained unchanged. Because protein expression change of claudin-1 alone seemed unlikely to fully explain the drop in epithelial resistance, we looked for other MC-LR–dependent pathomechanisms by analyzing TJ morphology by super-resolution STED microscopy: in contrast to conventional confocal laser-scanning microscopy, STED microscopy can resolve even subtle cytopathogenic effects beyond the subcellular redistribution of TJ proteins.

**FIGURE 3:**
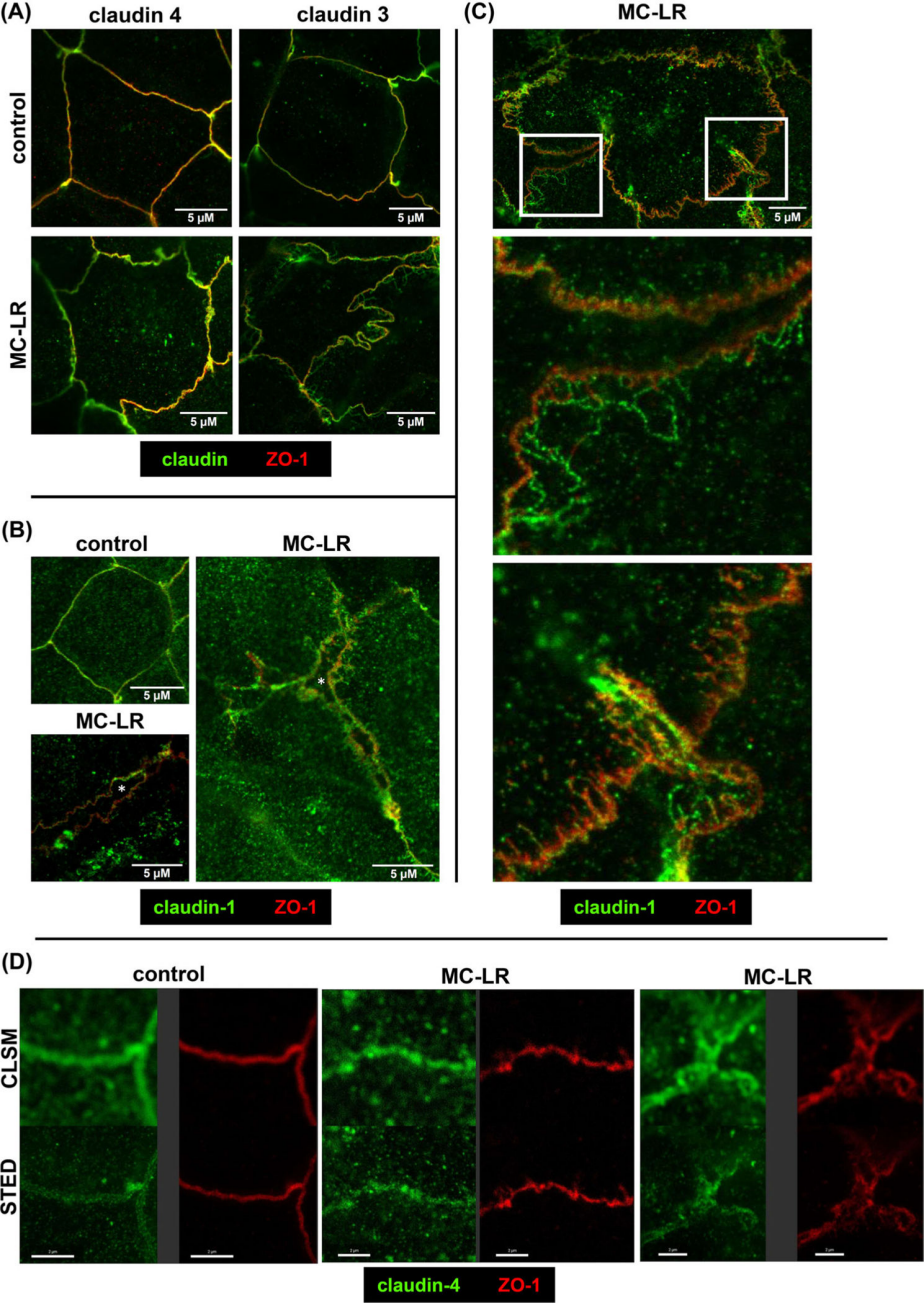
Super-resolution analysis of Caco-2 monolayers via immunofluorescence staining and STED microscopy 24 h after microcystin treatment. Super-resolution STED microscopy was performed on small intestine-like Caco-2 monolayers treated with 50 μM MC-LR for 24 h. Microscopic signals of zonula occludens protein-1 (ZO-1) are shown in red. Claudin-1, −3, and −4 were green colored. After MC-LR treatment, STED micrographs show (A) tight junction undulation and fraying in claudin-4 and claudin-3 staining or (B) split up of tight junctions at bicellular connections in claudin-1 staining. Asterisks indicate intercellular spaces (after retraction of lateral membranes). In the microscopic images, some cells appear larger after MC-LR treatment, as cell size increased by ~39% overall after counting cell numbers per overview. Bar = 5 μm. (C) Digital zoom (white squares) on MC-LR-treated cells via STED microscopy reveals finer details (middle and lower images). (D) Comparative images of conventional confocal laser-scanning microscopy (CLSM) imaging and STED imaging. The changes in ZO-1 microscopic signals compared to controls (left) ranged from slight wave-shape phenotype (middle) to more intense fraying (right) of the TJ in MC-LR-treated monolayers. The claudin-4 staining additionally showed a weaker signal in the TJ domain, indicating a discontinuous TJ pattern or even a redistribution of claudin off the TJ. Bar = 2 μm.

With both laser-scanning and STED microscopy, a redistribution of claudin-1 and −4 off the TJ toward intracellular aggregates was visible in MC-LR-treated cells (Figure 3). In addition, with STED microscopy, more cytopathogenic changes were observed, including fraying and undulating of the bicellular TJ domains of the epithelial cells, as well as the separation of TJs along lateral membranes, leading to cell detachment. The “split cell” phenotype was sporadically distributed in the observed areas of treated monolayers, whereas no detached cells were found in control cells.

Fraying and undulations of bicellular TJs were observed after microcystin exposure based on the distributions of ZO-1, claudin-3, and claudin-4 (Figure 3A). Claudin-3 was redistributed into a mesh-like network at lateral membranes. This also suggests that microcystin may have cytopathic effects on the cytoskeleton and that this may be the mechanism underlying the morphologic changes observed. An example of complete retraction at a bicellular TJ is depicted in the staining of ZO-1 together with claudin-1 (Figure 3). Thus, we depicted the pathogenic action on barrier function in a fraying TJ stage, before TJ split up and the separation of cell membranes of adjacent cells (Figure 3C). Undulating TJs were displayed in detail in respect to the comparison between CLSM and STED imaging (Figure 3D). In microscopic overviews (125 × 125 μm) ~60–80 cells per frame were counted of which 3.3 ± 0.6% showed undulations of the TJ in controls, whereas after treatment with MC-LR, 52.5 ± 2.2% of the cells showed the undulating phenotype (*p* < 0.001, *n* = 5).

### Epithelial apoptosis induction by microcystin

**FIGURE 4:**
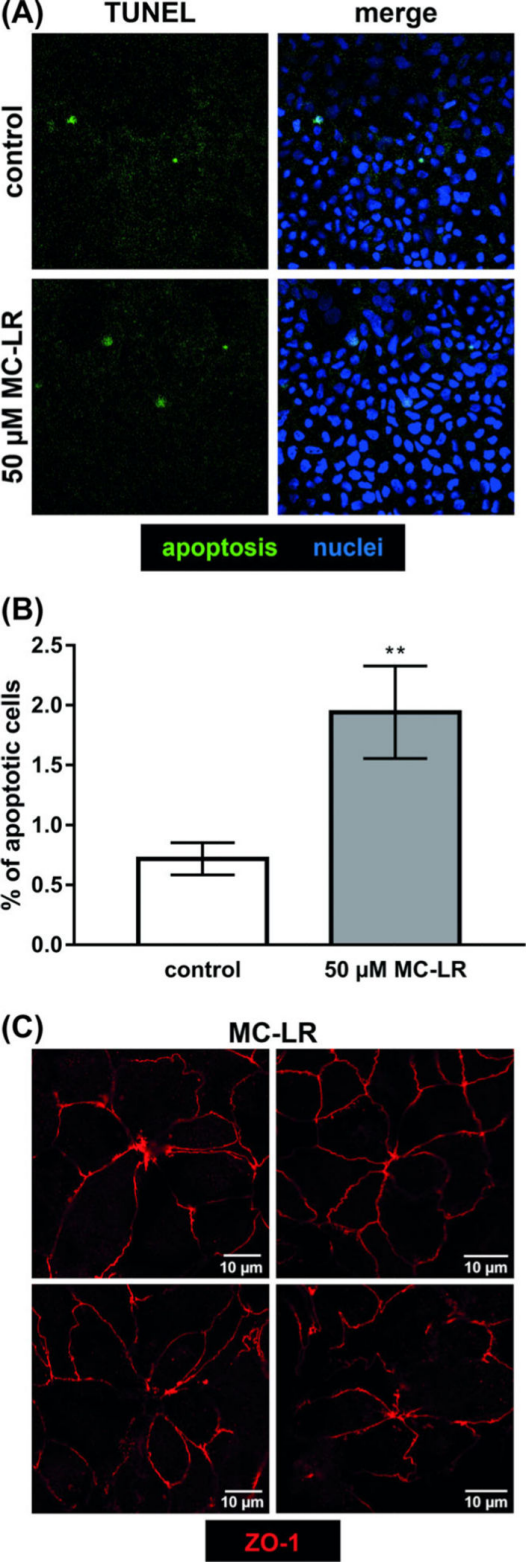
Apoptosis induction by microcystin-LR. (A) Small intestine-like Caco-2 monolayers were treated with 50 μM MC-LR. After 24 h, the rate of apoptotic cells was evaluated using TUNEL staining and LSM. TUNEL-positive cells are colored in green. Nuclei were stained with DAPI in blue. (B) Counting of apoptotic cells and all visible nuclei in a low-power field revealed the apoptotic ratio, each monolayer was counted in five random low-power fields; *n* = 3, **p < 0.01, *t*-test with Welch’s correction. (C) Four examples of restitutional rosette formation in MC-LR-treated cell monolayer. ZO-1 is colored red. Bar = 10 μm.

The physical separation between cells raises the possibility of cytotoxicity. We, therefore, evaluated cell death, which has been shown to either have no effect or to reduce epithelial barrier function, depending on the specific experimental system. TUNEL demonstrated a modest increase in cells with fragmented DNA (Figure 4A), from 0.7% in control monolayers to 1.8% apoptotic cells in MC-LR-treated monolayers (Figure 4B). However, the pan-caspase inhibitor Q-VD-OPh was unable to prevent MC-LR-induced TER loss over 24 h with 84 ± 3% of the initial TER in MC-LR versus 88 ± 4% in MC-LR and Q-VD-OPh-treated cells (n.s., *n* = 5, *p* = 0.40). Notably, rosette formation at sites of cell extrusion confirms the presence of apoptotic events in MC-LR-treated monolayers as well as intact restitution responses during extrusion (Figure 4C).

### Cytoskeletal disorganization leads to TJ disruption

**FIGURE 5:**
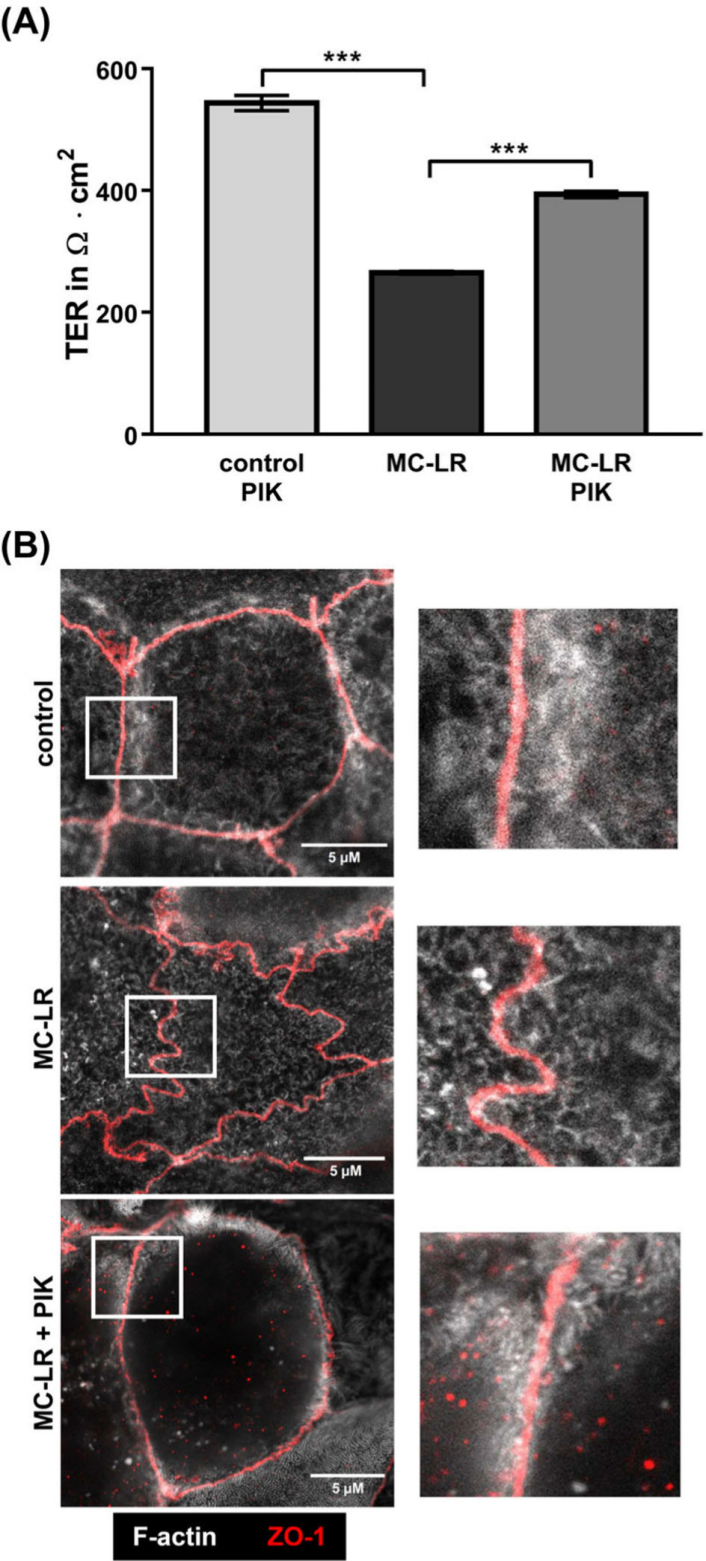
Recovery of epithelial barrier function and microstructural analysis of Caco-2 monolayers via TER measurement and immunofluorescence staining and STED microscopy after MC-LR treatment. (A) Inhibitor experiment. The specific MLCK inhibitor PIK had a significant effect in restoring barrier function 18 h after MC-LR treatment. All groups started with equal TER values, and the MC-LR group was different from untreated control with *p* < 0.001; *n* = 4, **p < 0.01, *t*-test with Welch’s correction. (B) Microscopic findings. Cells showed more condensed F-actin (white) after 24 h of MC-LR treatment. ZO-1 is colored red and showed undulation of the TJ in the treated cell monolayers. Undulation of ZO-1 could be mitigated by the addition of 200 μM D-reverse PIK (PIK), while the condensation of F-actin appears weaker. Depicted is one cell per condition in the left row (bar = 5 μm) and white squares with respective detail images (digital zoom) in the right row.

The MLCK inhibitor PIK is capable to restore normal TJ profiles. The stable PIK analog DrPIK could protect monolayers from MC-LR-induced barrier loss and restore normal TJ morphology. Although incomplete, DrPIK markedly attenuated MC-LR-induced barrier loss (Figure 5A). More strikingly, DrPIK restored smooth TJ F-actin and ZO-1 profiles (Figure 5B). Thus, the significant fraction of the barrier loss induced by MC-LR reflects cytoskeletal TJ regulation.

## DISCUSSION

### Health risks

Rising water temperatures due to climate change exacerbate algal blooms. During these events, cyanobacteria (blue-green algae) produce phosphatase inhibitors, such as MC-LR, in response to high phosphate concentrations present and polluted water.^10–12^ This may represent one mechanism by which water contamination by blue-green algae is harmful to humans, animals, and the aquatic life. Livestock animals (e.g., cattle) and pets (e.g., dogs) are especially at risk of poisoning from cyanotoxins.^13^ Fatalities, especially in dogs, have been reported following cyanobacterial poisoning with microcystins,^14^ and the microcystin LD_50_ has been calculated to be ~50 μg/kg bodyweight.^15^ Symptoms induced by sublethal cyanotoxin exposure include irritation of skin, eyes, nose, throat, and lungs, as well as neurological symptoms. Most relevant, however, may be the intestinal disease following the ingestion of cyanotoxins or contaminated water, which can lead to abdominal pain, vomiting, diarrhea, and liver damage.

The intestinal pathology induced by ingested MC-LR begins with the disruption of epithelial integrity. This barrier loss as well as MC-LR uptake into the mucosa leads to subepithelial inflammatory responses. Finally, exposure of the liver via the portal circulation can result in severe hepatotoxicity. Here, we focused on the mechanisms of MC-LR-induced barrier loss. Our studies indicate that barrier loss occurs by at least two separate mechanisms reflecting cytoskeletally mediated increases in TJ leak pathway permeability and epithelial damage that results in unrestricted pathway permeability increases. The latter reflects, in part, cell death, but the mechanisms by which MC-LR induces cell death remain to be defined.

### Epithelial barrier dysfunction

We were able to show that barrier-forming claudins are affected, which could explain part of the barrier disruption in Caco-2 cell monolayers but is unlikely to explain increased flux across the leak pathway. Previous studies have characterized this barrier MC-LR-induced barrier loss in terms of TER measurements and reduced the expression of occludin and ZO-1.^16,17^ Specific claudins, however, have not been previously studied. Here, we focused on the main barrier-forming claudins expressed in Caco-2 cells. Claudin-1 protein expression was reduced, which could contribute to the functional barrier impairment. However, claudins were only rarely seen to be redistributed away from TJs. Membrane undulations and cell loss were far more prominent, suggesting that cytoskeletal TJ regulation and cytotoxicity were more significant mechanisms of MC-LR-induced barrier loss. Both of these mechanisms of barrier loss can lead to watery diarrhea by the leak flux mechanism.

### Protein phosphatase inhibition and changes of the cytoskeleton

This view on the phenomenon of TJ proteins being pulled out of the TJ domain of the enterocytes is quite mechanical, but it is in line with cell signaling regulation (e.g., MLCK pathway). Cortical actin (cortactin) also known as perijunctional actomyosin is highly regulated by phosphorylation and phosphatases. The interaction of cortical actin with the accessory TJ protein ZO-1 then leads, when constricted, to undulation of the membrane (due to actomyosin contraction) and finally to the retraction of claudins. In this way, the tension on ZO-1 and claudins led to the observed fraying and undulation of TJs, revealed by super-resolution microscopy. This is plausible for the action of MC-LR through phosphatase inhibition, which is followed by overphosphorylation of proteins in the cells and prevents MLC dephosphorylation. This allows unopposed activity of MLCK and hyperphosphorylation of MLC. In our STED micrographs of MC-LR-treated cells, the perijunctional F-actin shows up brighter than in control staining, reflecting a reticular condensation, which points to a constricted stage of the cytoskeleton causing the undulation of TJs. Previous work has shown that TJ membrane undulations (syn. ruffling) are a marker of increased MLCK phosphorylation.^18^ Concomitantly to the TJ undulation, claudin-1 expression was decreased and the permeability for small macromolecules was increased,^18^ similarly to our findings. We, therefore, hypothesized that, through its activity as a phosphatase inhibitor, MC-LR inhibited myosin phosphatase and allowed unopposed action of MLCK and increased MLC phosphorylation. To test this hypothesis, we used a specific MLCK inhibitor, DrPIK, and found that it was able to markedly attenuate MC-LR-induced barrier loss.

In contrast, the perijunctional cytoskeleton can also be affected by bacterial pore-forming toxins (PFTs), but via different signaling pathways. The PFTs integrate into the apical membrane of the host cell and form pores, conductive for, for example, potassium and calcium. In a previous study, aerolysin, a PFT from *Aeromonas hydrophila*, induced a calcium influx in intestinal HT-29/B6 epithelial cells followed by calcium signaling, which then led to actomyosin constriction via MLCK activation and resulted in a massive redistribution of TJ proteins.^19^

Beyond MLC, as an inhibitor of PP1 or PP2A phosphatases, MC-LR can affect phosphorylation-dependent signaling via protein kinase C and other transduction pathways. Moreover, MC-LR may, indirectly, allow increased phosphorylation of claudins, which has been reported to reduce barrier function by inhibiting protein kinase A and serine/threonine-specific protein kinase activated by GTP-bound RhoA (ROCK).^20^ The failure of DrPIK to fully restore barrier function is consistent with the modest increase in TUNEL positivity following MC-LR treatment and suggests that cell death is a second pathologic mechanism. This may reflect MC-LR toxicity due to hyperactivation of phosphorylation-dependent processes. Accordingly, we demonstrated modest increases in cell death after MC-LR treatment. In principle, apoptosis induction is barrier-relevant.^21^ However, the pan-caspase inhibitor Q-VD-OPh was unable to limit barrier loss induced by MC-LR. Moreover, we observed rosette formation. This suggests that either apoptosis was not the primary mechanism of cell death or, alternatively, that, in this model, single cell apoptosis events were rapidly closed via purse-string contraction and had little effect on barrier function.^22,23^ Nevertheless, both TUNEL and compensatory cell spreading, that is, restitution, indicate that MC-LR did induce cell loss.

Interestingly, previous work has suggested that barrier loss induced by okadaic acid, the toxin responsible for diarrheic shellfish poisoning that inhibits PP1 and PP2A, is unaffected by MLCK inhibition with ML-9.^24^

Since the MLC is substrate of PP1, the phospho-status of the affected cells may be higher after the inhibition of PP1 by MC-LR.^25^ PP1 and PP2A can interact with over 50 target proteins in the cell, regulating multiple cell functions, including actomyosin contraction or microtubule stability.^26–28^ PP2A is the major phosphatase for microtubule-associated proteins (e.g., CDC2 and CDC25), as well as Raf, MEK, and Akt.^29^

Microtubule, intermediate filaments, and actin show morphological alterations within 20 min after MC-LR treatment (10–200 μM) in hepatocytes and fibroblasts.^30^ Thus, the opening of the leak pathway in our Caco-2 monolayers may mainly depend on the extent of the alterations in the actin cytoskeleton.

## CONCLUDING REMARKS

We have shown that even at low effective, MC-LR induces cytoskeletal TJ regulation. At higher concentrations, MC-LR leads to cell death.^31^ MC-LR is released by *Microcystis* spp. when the bacterial membrane bursts. High local MC-LR concentrations can, therefore, follow ingestion of intact cyanobacteria in their rupture adjacent to the mucosal surface. However, the bacteria could be killed within the stomach, so that free MC-LR is released into larger volumes and toxin concentrations are lower. We, therefore, speculate that, in vivo, low MC-LR concentrations within the small intestine are responsible for TJ barrier loss, while higher concentrations are responsible for cell death. Concentration within the portal circulation may also lead to high-dose-induced hepatotoxicity.

In conclusion, MC-LR induces constriction of the perijunctional cytoskeleton in a small intestinal Caco-2 cell followed by the disruption of TJs, which leads to epithelial barrier dysfunction. This leak flux mechanism by opening the leak pathway can explain the watery diarrhea that occurs in humans following the ingestion of *Microcystis*-contaminated water.
